# Macrophages, Low-Grade Inflammation, Insulin Resistance and Hyperinsulinemia: A Mutual Ambiguous Relationship in the Development of Metabolic Diseases

**DOI:** 10.3390/jcm11154358

**Published:** 2022-07-27

**Authors:** Gerhard Paul Püschel, Julia Klauder, Janin Henkel

**Affiliations:** 1 Institute of Nutritional Science, Department of Nutritional Biochemistry, University of Potsdam, D-14558 Nuthetal, Germany; manowsky@uni-potsdam.de; 2 Faculty of Life Sciences: Food, Nutrition and Health, Department of Nutritional Biochemistry, University of Bayreuth, D-95326 Kulmbach, Germany

**Keywords:** NAFLD/MAFLD, type 2 diabetes, obesity, vicious cycle, TLR signaling, M1/M2 differentiation, Akt pathway

## Abstract

Metabolic derangement with poor glycemic control accompanying overweight and obesity is associated with chronic low-grade inflammation and hyperinsulinemia. Macrophages, which present a very heterogeneous population of cells, play a key role in the maintenance of normal tissue homeostasis, but functional alterations in the resident macrophage pool as well as newly recruited monocyte-derived macrophages are important drivers in the development of low-grade inflammation. While metabolic dysfunction, insulin resistance and tissue damage may trigger or advance pro-inflammatory responses in macrophages, the inflammation itself contributes to the development of insulin resistance and the resulting hyperinsulinemia. Macrophages express insulin receptors whose downstream signaling networks share a number of knots with the signaling pathways of pattern recognition and cytokine receptors, which shape macrophage polarity. The shared knots allow insulin to enhance or attenuate both pro-inflammatory and anti-inflammatory macrophage responses. This supposedly physiological function may be impaired by hyperinsulinemia or insulin resistance in macrophages. This review discusses the mutual ambiguous relationship of low-grade inflammation, insulin resistance, hyperinsulinemia and the insulin-dependent modulation of macrophage activity with a focus on adipose tissue and liver.

## 1. Introduction

The growing prevalence of overweight and obesity in humans is the consequence of the encounter of a genetic background shaped by the need for high resilience against longer phases of starvation and strenuous physical activity with an environment that is characterized by an affluent food supply and sedentary lifestyle [1]. Overweight and obesity pose a problem because they are risk factors for potentially fatal metabolic diseases. In the long run, overweight and obesity are characterized by (1.) dysfunction of white adipose tissue [2], the major physiological site for storage of excess energy, (2.) ectopic lipid accumulation that manifests, among others, as metabolic dysfunction-associated fatty liver disease (MAFLD) [3], (3.) chronic, low-grade inflammation [4] as well as (4.) insulin resistance and hyperinsulinemia [5] that are mutually interlinked [6,7] and, at later stages, may progress to type 2 diabetes. Resident tissue macrophages play a pivotal role in the maintenance of normal tissue homeostasis, yet both functionally altered resident macrophages and newly recruited monocyte-derived macrophages are also key players during the development of low-grade inflammation [8]. While low-grade inflammation certainly is a driver in the progression of whole-body insulin resistance, metabolic dysregulation, insulin resistance and hyperinsulinemia contribute to the development and progression of low-grade inflammation by altering macrophage functions [9]. The current review does not intend to provide a comprehensive summary of research in this field, but rather tries to give an idea of the complex egg-and-hen problem in insulin resistance, hyperinsulinemia and inflammation using selected examples in liver and adipose tissue that show the mutual interrelation.

## 2. Insulin Signaling and Mechanisms of Insulin Resistance

Here, only a very short introduction to insulin signaling and the molecular basis of insulin resistance is provided. This does not exceed extended textbook knowledge. For more detail, the reader is referred to several excellent and comprehensive recent reviews [10,11,12,13,14,15,16]. Readers familiar with this topic may skip this section.

Insulin is a key regulator of metabolic functions in skeletal muscle, white adipose tissue and liver. It acutely enhances glucose uptake into skeletal muscle, which is the main site of glucose disposal after meals, by stimulating the translocation of the glucose transporter 4 (Glut4) from an intracellular vesicular storage compartment to the plasma membrane and augmenting glycogen synthesis and glucose oxidation [17,18,19]. In white adipose tissue, insulin enhances glucose and fatty acid uptake, stimulates triglyceride synthesis and at the same time, and probably more importantly, it inhibits lipolysis by activating phosphodiesterase and phosphoprotein phosphatases thereby antagonizing the adrenalin-dependent activation of lipolytic enzymes [20,21]. In the liver, insulin stimulates aerobic and anaerobic glucose metabolism and the incorporation of glucose into glycogen. It inhibits glucose production directly by inactivating glycogen phosphorylase and by inhibiting and repressing gluconeogenic enzymes [22,23,24]. In addition, it inhibits hepatic gluconeogenesis indirectly by suppressing protein degradation in skeletal muscle, thereby reducing the flux of gluconeogenic substrates to the liver, and by inhibiting lipolysis in adipocytes, thereby reducing the flux of fatty acids to the liver, the oxidation of which provides reduced co-enzymes and the energy for gluconeogenesis [22,25].

**Figure 1:**
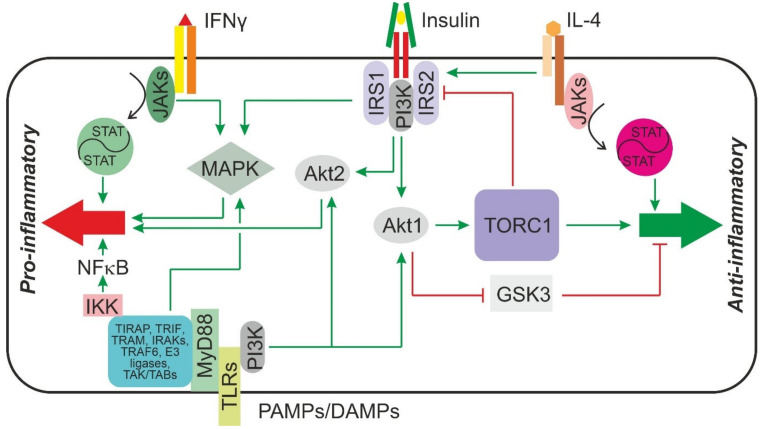
**Crosstalk between insulin receptor, toll-like receptors (TLRs), interleukin-4 (IL-4) and interferon γ (INFγ) signaling in macrophages**. The activated insulin receptor tyrosine kinase serves as docking site for insulin receptor substrates (IRS) and downstream signaling components such mitogen-activated kinases (MAPK), phosphoinositide-3-kinases (PI3K) and protein kinase B (PKB)/Akt kinases that modulate the activity of TORC1 and GSK3. The insulin receptor signal chain shares signal chain elements with the other receptors: Toll-like receptors (TLRs) recognize pathogen-associated molecular patterns (PAMPs) or damage-associated molecular patterns (DAMPs). Upon ligand binding they recruit adaptor proteins, e.g., Myeloid differentiation primary response 88 (MyD88), TIR domain-containing adapter protein (TIRAP), TIR-domain containing adapter-inducing interferon-β (TRIF) and TRIF-related adaptor molecule (TRAM), and activate downstream components such as IL-1 receptor-associated kinases (IRAKs), TNF receptor-associated factor 6 (TRAF6), ubiquitin E3 ligases and a TAK/TAB kinase (TGF-β-activated kinase/TAK binding protein) complex in the canonical signal chain. This results in the activation of the inhibitor of kappa B kinase (IKK) complex and activation of the transcription factor nuclear factor kappa B (NFκB) inducing the gene transcription of pro-inflammatory cytokines and IFNγ. Next to NFκB, MAPK and Akt2, which TLRs share with the insulin receptor signaling chain, stimulate the transcription of pro-inflammatory mediators. Interferon γ signaling by activation of the interferon receptor γ (IFNRG) activates janus kinases (JAKs) and signal transducers and activators of transcription (STAT) 4 and 1 as downstream signaling molecules. In addition, it activates MAPKs triggering the pro-inflammatory polarization of macrophages. MAPKs are a potential site of synergistic crosstalk with insulin. Interleukin-4 (IL-4)-mediated signaling activates STAT6, which contributes to the differentiation of the M2 macrophage phenotype. Insulin can act in concert by an Akt1-dependent activation of target of rapamycin complex 1 (TORC1) and inhibition of glycogen synthase kinase 3 (GSK3) providing anti-apoptotic and anti-inflammatory signals. In addition, IL-4 causes an activation of IRS2 and its downstream signaling describing a potential crosstalk between IL-4 and insulin signaling that may drive M2 macrophage differentiation.

All these actions of insulin are mediated by the insulin receptor, a hetero-tetrameric receptor tyrosine kinase that is activated by insulin binding. The activated tyrosine kinase domains cross-phosphorylate tyrosine residues in the opposite intracellular domain, generating phospho-tyrosine docking sites for proteins with phospho-tyrosine binding domains [26]. For the insulin receptor, the first relay proteins in this signal chain are the tissue-specifically expressed insulin receptor substrates (IRS), which get phosphorylated at tyrosine residues themselves [27]. The tyrosine-phosphorylated IRS then recruits more downstream signaling components that activate different signal chains (see Figure 1), leading to the activation of mitogen-activated kinases (MAPK), in particular p44/42 extracellular signal-regulated kinases 1/2 (ERK1/2), the class 1A phosphoinositide-3-kinases (PI3K)—protein kinase B (PKB)/Akt kinase axis, as well as to a rise in cytoplasmatic Ca^2+^ and the production of reactive oxygen species, which at low concentrations enhance insulin signaling by inhibiting lipid (PTEN) and protein (PTP) phosphatases [28]. PI3K converts 4,5-phosphatidyl-inositol-bisphosphate (PI2) into 3,4,5-phosphatidylinositol-trisphosphate (PI3). Newly generated PI3 serves as a plasma membrane assembly site for a complex of PI3-dependent kinase, Akt kinase and the target of rapamycin complex 2 (TORC2) complex. PI3-dependent kinase and TORC2-kinase phosphorylate Akt kinase at Thr^308^ and Ser^473^, respectively, thereby activating the kinase [22,29]. Activated Akt kinases subsequently mediate many of the metabolic effects of insulin by phosphorylating its target proteins, for example glycogen synthase kinase 3β (GSK3β, disinhibition of glycogen synthesis), Akt target of 160 kDa (AS160, stimulation of Glut4 translocation), forkhead box protein O transcription factors (FoxO, inhibition of gluconeogenesis), phosphodiesterase (antagonize glucagon and adrenalin signaling), tuberous sclerosis complex 2 (TSC2, stimulation of TORC1-dependent protein synthesis and autophagy) and phosphoprotein phosphatases (activation of several covalently modified enzymes involved in energy storage) [30,31].

Insulin resistance interrupts this signaling chain. Serine/threonine kinases may phosphorylate either the insulin receptor or insulin receptor substrates at serine or threonine residues and thereby either inhibit coupling to downstream signaling components or prevent the activating tyrosine phosphorylation of these proteins. In the case of the insulin receptor substrate—depending on the side of phosphorylation—enhanced proteasomal degradation may ensue [32,33,34]. Notably, activation of the insulin receptor itself may enhance IRS serine phosphorylation by ERK1/2. Poor fatty acid handling, which results in metabolic accumulation of diacylglycerol, may activate novel protein kinase C (PKC) members, PKCε or PKCθ, that either phosphorylate the insulin receptor at threonine residues or the insulin receptor substrates at serine residues [32,35]. Similarly, ceramides, which are either generated in inflammatory signaling chains or accumulate in lipid metabolism, can activate PKCδ, which also causes IRS-serine phosphorylation. In addition, ceramides can activate protein phosphatase 2A (PP2a), which dephosphorylates and inactivates Akt kinase [36]. Finally, several cytokine receptor signal chains and pattern recognition receptor signal chains, as well as oxidative stress and ER stress, result in the activation of c-Jun N-terminal kinase (JNK) and the inhibitor of kappa B kinase β (IKKβ) that in turn can serine-phosphorylate the IRS [37,38].

Tyrosine kinase-associated cytokine receptors that transduce their signal via janus kinases (JAKs) and signal transducers and activators of transcription (STATs) enhance the transcription of suppressors of cytokine signaling (SOCS, e.g., SOCS3). SOCS are phospho-tyrosine binding proteins that can bind to the tyrosine-phosphorylated insulin receptor and IRS, thereby interrupting the insulin receptor signal chain. Interleukin 6 (IL-6) and leptin are two prominent examples [39,40,41].

## 3. Macrophage Sub-Populations and Their Function

Macrophages are key players both in the maintenance of tissue homeostasis and inflammation. Macrophages present a very heterogeneous population of cells of the innate immune system that have in common the capacity to phagocytose material that they, by means of an array of cell surface receptors, recognize as non-self such as microbes, particular matter or dead cells. Immunologically, they can be identified by surface markers that are common to all macrophages, e.g., F4/80 in mice or EMR1 (ADGRE1) in humans [42] or cluster of differentiation (CD) 64 [43]. Classically, two major sub-populations of macrophages were identified. Upon stimulation with lipopolysaccharide (LPS) or interferon γ (IFNγ), macrophages differentiate into so-called M1 macrophages that are characterized by the expression of CD11c, CD40 and the production of pro-inflammatory cytokines such as interleukin (IL) 1α, IL-1β, IL-6 or tumor necrosis factor α (TNFα). In human and mouse macrophages, CD274, CD38 and CD319 have been claimed to be the most reliable M1 markers [44]. M1 macrophages also express high levels of inducible nitrogen oxide synthase (iNOS) and NADPH oxidase to produce reactive nitrogen and oxygen species that confer a strong bactericidal potency to this sub-population [45]. By contrast, differentiation of macrophages in the presence of IL-4, IL-10 or transforming growth factor β (TGFβ) yields M2 macrophages that are characterized by the expression of CD206 and the production of anti-inflammatory cytokines such as IL-10. They express high levels of arginase and have a higher capacity for phagocytosis [45] and allow resolution of the inflammation and wound healing.

Macrophages can also be subdivided according to their origin and differentiation in response to signals in the tissue environment they inhabit. The majority of tissue-resident macrophages are derived from yolk sack or fetal liver progenitors [46] with little contribution from bone-marrow-derived cells, however, the extent to which these latter cells contribute varies between different tissues [47]. Tissue-resident macrophages are long-lived and are replaced by local proliferation. They are shaped by the local tissue metabolic and cytokine environment and display tissue-specific characteristics that they also maintain when artificially transferred into a different tissue environment that they are unable to colonize [48]. Once the tissue-specific characteristics are acquired they are stabilized by epigenetic changes [47]. Long-lived tissue macrophages may also derive from circulating blood monocytes that are retained in the tissue and develop into differentiated tissue resident macrophages when exposed to tissue-specific differentiation cues. This becomes particularly relevant if replacement by local proliferation is insufficient, e.g., after specific experimental depletion of Kupffer cells or as a consequence of steatohepatitis-mediated liver damage (see paragraphs below). However, in other tissues there appears to be a physiological continuous replacement of resident macrophages of fetal origin by monocyte-derived resident macrophages over the lifespan of an individual in absence of prior tissue damage [49,50]. These long-term resident macrophages, which in the gut and liver are characterized by the presence of marked T cell immunoglobulin and mucin domain containing 4 (Timd4) [51,52], irrespective of their origin, must be distinguished from monocyte-derived macrophages that acutely infiltrate the tissue response to chemotactic cues. Typically, these latter cells are characterized by the presence of the surface marker lymphocyte antigen complex locus C1 (Ly6C1), C-C-motif receptor 2 CCR2 and high expression of CD11b and CD11c [47,53,54]. These newly recruited macrophages generally have an M1-typical signature. It has been hypothesized that an imbalance between local proliferation of resident macrophages that confer tissue repair on the one hand and continuous recruitment of fresh monocyte-derived macrophages and their excessive local proliferation that maintains a pro-inflammatory phenotype on the other hand might contribute to sustaining chronic inflammation [47]. However, the overlap between resident and M2 versus newly recruited and M1 is by no means complete.

### 3.1. Adipose Tissue Macrophages

Tissue resident macrophages differ between different tissues, and also within the same tissue resident macrophages form subpopulations with subtype-specific functions. Adipose-tissue-resident macrophages apparently can be subdivided into two subpopulations [8], vascular-associated macrophages (VAM) and lipid-associated macrophages (LAM). Vascular-associated macrophages are characterized by high expression lymphoid endothelium hyaluronan receptor 1 (Lyve1), low expression of major histocompatibility complex II (MHCII) and CD9. They most closely resemble the classical M2 macrophages in that they are positive for the mannose receptor CD206 and express high levels of the scavenger receptor CD163, Retnla (resistin-like α, Fizz-1) and the lectin CD209f [8]. The relative number of these vascular macrophages declines when adipose tissue expands whereas the relative number of lipid-associated macrophages increases.

Lipid-associated macrophages are characterized by high expression levels of CD9 and MHCII and low expression of Lyve1. The lipid-associated macrophages form crown-like structures around dysfunctional or dying adipocytes and clear the locally accumulated extracellular lipids released by dysfunctional or dying adipocytes. They express the receptor TREM2 (triggering receptor expressed on myeloid cells 2), which among others binds phospholipids and DNA that may be released from dying cells. In response to these ligands, TREM2 among others activates PI3K and Akt and calcium signaling in a DAP10/12-dependent manner, enhances phagocytosis of apoptotic cells and cellular debris, induces enzymes and transporters involved in lipid handling [55] and reduces the production of pro-inflammatory cytokines such as TNFα [56]. A recent study showed that TREM2 is not merely a marker of these cells, but TREM2 signaling is essential in triggering the differentiation into lipid-associated macrophages, the majority of which is derived from monocyte precursors [57]. These lipid-associated macrophages initially fulfill homeostatic functions in adipose tissue by clearing lipid-rich extracellular material and by preventing further adipocyte hypertrophy and thus may help to maintain insulin sensitivity.

Eventually, however, the excessive supply of dietary lipids results in disproportionate adipocyte hypertrophy as well as hyperplasia that is not accompanied by sufficient capillarization [14,58,59]. The resulting hypoxia activates cellular signaling pathways that lead to a stress response, such as the release of pro-inflammatory cytokines and immune cell-recruiting chemokines [11,60,61]. Due to the activation by these locally produced cytokines and other mediators such as saturated fatty acids or advanced glycation end products as well as circulating signals such as modified lipoproteins or endotoxins, macrophages acquire a more inflammatory phenotype. Additional monocyte-derived macrophages are recruited by the chemokines. As a result, the balance tips toward chronic inflammation and the cells contribute to the rendering of adipose tissue insulin resistant. The phenotype of these macrophages still differs from the classical M1 phenotype and the cells are best described as metabolically activated macrophages [8]. These macrophages differentiate from M0 macrophages in presence of high concentrations of palmitate, insulin and glucose, palmitate being the most relevant cue. While they express significantly higher levels of the pro-inflammatory cytokines TNFα, IL-1β and IL-6 than undifferentiated M0 macrophages, the expression level of these cytokines is lower than in classical M1 macrophages. In contrast to M1 macrophages, they express enzymes and transporters involved in lipid metabolism, similar to the lipid-associated macrophages. The expression of the classical M2 marker CD206 is low in these cells [44].

### 3.2. Liver Macrophages

Kupffer cells are resident macrophages with long projections located mainly on the luminal side of the sinusoidal endothelium in the liver [62]. They differentiate from erythromyeloid precursor cells of the yolk sac that migrate to the liver in early embryogenesis [63] and can self-renew and proliferate as needed [64]. Their principal physiological functions are the elimination of systemic and gastrointestinal pathogens and regulation of iron metabolism. They are also the first cells to respond to liver injury [54,65] such as non-alcoholic steatohepatitis (NASH). Profound changes within the liver macrophage pool have been described to occur in the course of NASH development [54,64,65,66]. Non-alcoholic fatty liver disease (NAFLD) is a multifactorial disorder with a complex pathophysiology including a multiple-hit hypothesis that defines the hepatic manifestation of the metabolic syndrome (reviewed in [67,68,69]). Due to the link between NAFLD and its comorbidities, the term metabolic dysfunction-associated fatty liver disease (MAFLD) has recently been proposed as a broader alternative covering the heterogeneous nature of this complex disease. Criteria of MAFLD are the presence of hepatic steatosis and one of the following three criteria, namely obesity, presence of type 2 diabetes mellitus or evidence of metabolic dysregulation [70,71].

Steatohepatitis (NASH) is the more severe form of NAFLD/MAFLD, where steatosis is accompanied by hepatic infiltration with inflammatory cells and progressive fibrosis [72,73]. Kupffer cells are F4/80^high^ CD11b^low^ CX3CR1^low^ in combination with the specific expression of the C-type lectin CLEC4F and the phosphatidylserine receptor Tim4 (CLEC4F^+^ Tim4^+^) [52,74,75,76]. During NASH development, at least two subpopulations with different transcriptional profiles and functions populate the liver next to resident Kupffer cells. These cells, in contrast to Kupffer cells, are F4/80^low^ CD11b^high^ CX3CR1^high^ in combination with CLEC4F^+^ or CLEC4F^−^ and Tim4^−^. Whereas Kupffer cells clear gut-derived LPS without reaction, recruited monocyte-derived macrophages are particularly sensitive to the LPS-dependent stimulation of pro-inflammatory cytokines [76].

As a consequence of liver damage caused by steatohepatitis, self-renewal of the Kupffer cells is impaired and the embryo-derived Kupffer cells are replaced by monocyte-derived Kupffer cells that differentiate in response to signals that originate from hepatic stellate cells, sinusoidal endothelial cells and hepatocytes [52,77], and acquire mature Kupffer cells markers such as TREM2, CD9, CD68 and CLEC4F, but lose their expression of Tim4 [52,57,78,79,80,81]. This macrophage subpopulation is also referred to as NASH-associated macrophages (NAMs) [82,83]. In mice with diet-induced NASH, transcriptome analysis has revealed that Kupffer cells lose some of their gene expression identity but augment the expression of TREM2 and CD9 [84]. High hepatic expression of TREM2 is strongly associated with disease severity reflecting a higher impact of steatosis, inflammation, hepatocyte ballooning and fibrosis, which are summarized in the NASH activity score [83].

The other main subpopulation formed by recruited macrophages that could be obtained from mice with diet-induced NASH, has been termed hepatic lipid-associated macrophages (hepatic LAMs) because of their gene signatures associated with lipid metabolism similar to LAMs from obese adipose tissue [57,79]. These macrophages express, among others, osteopontin, which is linked with the development of fibrosis, and are found in zones of the liver with a decreased number of resident Kupffer cells and an increased expression of desmin in a study with diet-induced NASH in mice [80]. These CLEC4F-negative macrophages, which replace Kupffer cells in NASH livers, also have a similar transcriptome to scar-associated macrophages in fibrotic human liver [85].

Whereas Kupffer cells, similar to resident macrophages of white adipose tissue, probably contribute to maintaining insulin sensitivity of the organ system, the newly recruited macrophages rather contribute to the development of insulin resistance.

It is important to keep in mind the large diversity of these different macrophage populations of different origin when comparing the seemingly inconsistent observations of how insulin affects macrophage function.

### 3.3. Signaling Chains Involved in Macrophage Polarization and Activation

Macrophages sense their environment by a number of plasma membrane or endosomal pattern and scavenger receptors, among others, the toll-like receptors (TLRs), which recognize pathogen-associated molecular patterns (PAMPs) or damage-associated molecular patterns (DAMPs). The polarization of macrophages is also shaped by cytokines, namely interferon γ (INFγ), IL-4, IL-10 or TGFβ. Lipid-associated macrophages (LAMs) sense the lipids in their environment by TREM2. All these receptors have their individual canonic signal chains, yet they all have in common that they share certain knots of the insulin receptor signaling chain. It has long been known that macrophages express insulin receptors and that insulin may affect macrophage function [86]. All the components of the insulin receptor signaling chain are active in macrophages [87,88]. Thus, it is likely that insulin can interfere with these signaling chains. It is beyond the scope of this review to discuss all receptor signaling chains in detail, but a few prominent examples that highlight the crossroads with insulin receptor signaling may serve as an illustration.

Toll-like receptors are platform-forming type 1 transmembrane protein receptors that upon ligand binding recruit adaptor proteins, e.g., myeloid differentiation primary response 88 (MyD88), TIR domain-containing adapter protein (TIRAP), TIR domain-containing adapter-inducing interferon β (TRIF) and TRIF-related adaptor molecule (TRAM) [89], to their cytoplasmic TIR (Toll/interleukin-1 receptor) domain (Figure 1). In the case of TLR4, MyD88 and TIRAP then assemble and activate the downstream components of the canonical signal chain, namely several IL-1 receptor-associated kinases (IRAKs), which by the mediation of TNF receptor-associated factor 6 (TRAF6) and ubiquitin E3 ligases activate a TAK/TAB kinase complex, which in turn phosphorylates the inhibitor of kappa B (IκB) kinase complex resulting in the activation of the transcription factor nuclear factor kappa B (NFκB) by phosphorylating the inhibitor of kappa B (IκB) and causing its degradation. In addition, the TAK/TAB kinase complex can activate the MAPKs extracellular signal-regulated kinases 1/2 (ERK1/2) and JNK1/2, the former of which can also be activated in a ras-RAF-MEK1/2-dependent manner by TLR4 [90,91]. Activation of MAPK and NFκB induces the transcription of the genes for pro-inflammatory cytokines and IFNγ. By a different, MyD88-independent signaling chain that results in the activation of IRF3, the genes of type I interferons and IL-10 are induced. TLRs, namely TLR4 [92] and TLR2 [93], can also activate the PI3K-Akt-signaling pathway (Figure 1). Activation of Akt1 but not Akt2 results in an increase in IL-10 formation and repression of the pro-inflammatory cytokine IL-12 [93]. Notably, ablation of Akt2 does not merely leave the cytokine production unaffected, but rather further decreases the production of pro-inflammatory cytokines and enhances the production of IL-10 and arginase-1. By contrast, ablation of Akt1 increases the production of pro-inflammatory cytokines [94]. Thus, Akt1 activation rather drives M2 differentiation whereas Akt2 activation drives M1 differentiation [95]. Both Akt1 and Akt2, as well as MAPK, are downstream elements of the insulin receptor signal chain (Figure 1). Therefore, it appears to be feasible that insulin, by activating Akt1, might attenuate the pro-inflammatory response of macrophages whereas by activating MAPK or Akt2 it might enhance it.

IL-4 signals through a tyrosine kinase-associated receptor and tyrosine-phosphorylates and activates STAT6. In addition, IL-4 causes tyrosine phosphorylation of IRS2 and downstream activation of PI3K and Akt kinase. IL-4 can signal through two different tyrosine kinase-associated receptor complexes. One of the complexes consists of IL-4Rα and IL-13-Rα, and the other of IL-4Rα and a γ-chain. While both complexes activate STAT6 signaling to a similar extent, only the IL-4Rα/γ-chain complex results in a strong IRS2 phosphorylation. While Akt-activation provides anti-apoptotic signals and in concert with STAT6 activation contributes to the differentiation of the M2 macrophage phenotype, it also inhibits IRS2 signaling in a TORC1-dependent negative feedback loop [96]. Since macrophages express the insulin receptor and can tyrosine-phosphorylate IRS2 after stimulation with insulin, it appears to be possible that both a positive or a negative crosstalk may exist between insulin and IL-4 signaling (Figure 1).

Interferons are potent regulators of macrophage function. While all type 1 interferons signal through the interferon receptor α (IFNRA) and tend to shift macrophages towards an M2 polarization, the only type 2 interferon, interferon γ, signals through the interferon receptor γ (IFNRG) and triggers an M1 polarization of macrophages. Both receptors are tyrosine kinase-associated receptors that, upon ligand binding, recruit tyrosine kinase 2 (Tyk2) and JAK1 to activate STAT2 and STAT1 in the case of IFNRA or JAK1 and JAK2 to activate STAT4 and STAT1 in the case of IFNRG. In addition to these canonical pathways, both receptors have also been shown to activate signals downstream of PI3 kinase as well as MAPK [97], thus allowing for potential crosstalk with insulin receptor signaling (Figure 1).

The polarization and activity of macrophages are not only controlled by cytokines and receptor-dependent mechanisms, but also by changes in their metabolism (See [97,98,99] for recent reviews). Among several intermediary metabolites that may directly regulate macrophage differentiation, succinate favors M1 polarization of macrophages. Circulating succinate levels have been shown to increase in obese individuals or type 2 diabetic patients. Hypoxia and hyperglycemia independently elevated succinate release in adipose tissue of mice [100]. In addition to endogenous production, succinate may be derived from an altered gut microbiota in obese patients [101]. Succinate concentration has been shown to increase in macrophages upon LPS stimulation and then can trigger the expression of the pro-inflammatory cytokine IL-1β by stabilizing the transcription factor hypoxia-induced factor 1α (HIF1α) [102]. Interestingly, insulin has also been shown to stabilize HIF1α by an Akt-dependent inhibition of GSK3β, which phosphorylates HIF1α and thereby causes its association with an ubiquitin–ligase complex and proteasomal degradation (reviewed in [103]). Hence, also by this mechanism insulin could potentially modulate macrophage polarization and activity.

## 4. Role of Macrophages in the Development of Insulin Resistance: Inflammation as a Cause of Insulin Resistance

Several lines of evidence suggest that a chronic, low-grade inflammation in which tissue macrophages are central players is a cause of insulin resistance (see recent reviews [4,13,104]).

### 4.1. Adipose Tissue

With some reservation, adipose tissue may be considered the primary site of the development of chronic low-level inflammation in insulin resistance.

Expansion of adipose tissue in overweight and obese patients is correlated with an increase in macrophage content in this tissue [105,106]. In mouse models of obesity, the number of macrophages in adipose tissue was elevated and positively correlated both with the body mass and the adipocyte size in white adipose tissue [107]. The increase in macrophage number and activity in adipose tissue has been associated with insulin resistance [108]. Macrophages appear to be the principal source of pro-inflammatory cytokines such as IL-1β and TNFα that can confer insulin resistance in insulin-sensitive cells [38,109,110]. In inflamed adipose tissue, interleukin production is further enhanced by a paracrine feed-forward loop involving CD4^+^ T cells producing IL-17 and IL-22 [111]. The increase in macrophage content is based on three processes, proliferation of resident macrophages [112,113], chemotactic recruitment of bone-marrow-derived macrophages and enhanced retention of macrophages in adipose tissue [13].

Inhibition of the recruitment of monocyte-derived macrophages by gene silencing of different receptors or ligands involved in chemotaxis-attenuated diet-induced insulin resistance [114,115,116], while over-expression of monocyte chemoattractant protein 1 (MCP1), a macrophage chemotactic protein that is also named C-C-Motif ligand 2 (CCL2), in adipose tissue increased the recruitment of macrophages and exacerbated obesity-induced insulin resistance in mice [117]. Conversely, a deficiency in macrophage migration inhibitory factor (MIF), a pro-inflammatory factor with chemokine-like properties, reduced high-fat diet-mediated M1 macrophage infiltration in white adipose tissue compared to wild-type mice and resulted in a partial protection from diet-induced insulin resistance by alleviating tissue inflammation in MIF-deficient mice compared to wildtype littermates [118]. In line with the assumption that IL-1β secreted by the infiltrating macrophages is a major cause of insulin resistance, a lack of the IL-1 receptor I protected mice from high-fat diet-induced inflammation in adipose tissue and improved glucose homeostasis, although the number of macrophages was comparable between the genotypes [119]. Furthermore, a role of complement proteins and their receptors are discussed as regulator of macrophage activation and polarization. Both complement anaphylatoxin C3a and C5a receptors (C3aR, C5aR) are upregulated in obese adipose tissue of high-fat diet-fed rodents [120,121]. Elimination of the C3a receptor reduced macrophage recruitment into adipose tissue and thereby improved insulin sensitivity both on standard and on high-fat diet [122]. Similarly, a deficiency in C5a receptor was associated with reduced accumulation of total and M1 macrophages in adipose tissue in mice with diet-induced obesity [121]. This was accompanied by an increased secretion of IL-10 and improved insulin sensitivity in adipose tissue compared to wildtype mice fed a high-fat diet [121]. The role of C3aR and C5aR was confirmed by another study that used receptor-selective antagonists of either C3aR or C5aR in obese rats. Inhibition of C3aR- or C5aR-mediated signaling reduced adipose tissue inflammation and resulted in improved glucose tolerance and reduced insulin resistance compared to controls [120].

The enhanced retention of macrophages was in part mediated by an upregulation of netrin-1 expression, a secreted laminin-related molecule with neuroimmune guidance function, in adipose tissue [123]. Macrophage-specific elimination of netrin-1 resulted in an improved glucose tolerance in high-fat diet-fed mice and in an about 50% reduction in the macrophage number in visceral adipose tissue with only a modest impact on adiposity or fat cell size [124]. Notably, however, apart from reducing the macrophage number, macrophage-specific elimination of netrin-1 resulted in a phenotype switch in the macrophages, improving the capacity for lipid handling. Thus, the observed impact on glucose tolerance cannot unequivocally be attributed to the reduction in macrophage number.

In addition, experiments eliminating adipose tissue macrophages indicate that there is not merely a correlation between macrophage accumulation in adipose tissue and systemic insulin resistance but a causal relationship. Elimination of visceral white adipose tissue macrophages by intraperitoneal injection of clodronate liposomes reduced diet-induced insulin resistance. While the improvement in insulin sensitivity in animals that received clodronate liposomes during the entire dietary intervention could largely be attributed to the reduced accretion of fat mass, clodronate liposome injection at a later time point of the dietary intervention still improved insulin sensitivity but only mildly affected the diet-induced weight gain [125]. In a similar study, injection of clodronate liposomes in mice with diet-induced obesity caused an improvement in insulin sensitivity with a marked increase in insulin-mediated suppression of hepatic glucose production during an euglycemic hyperinsulinemic clamp. No differences in weight gain or fat mass between control and clodronate-liposome-treated animals were observed [126].

### 4.2. Liver

**Figure 2:**
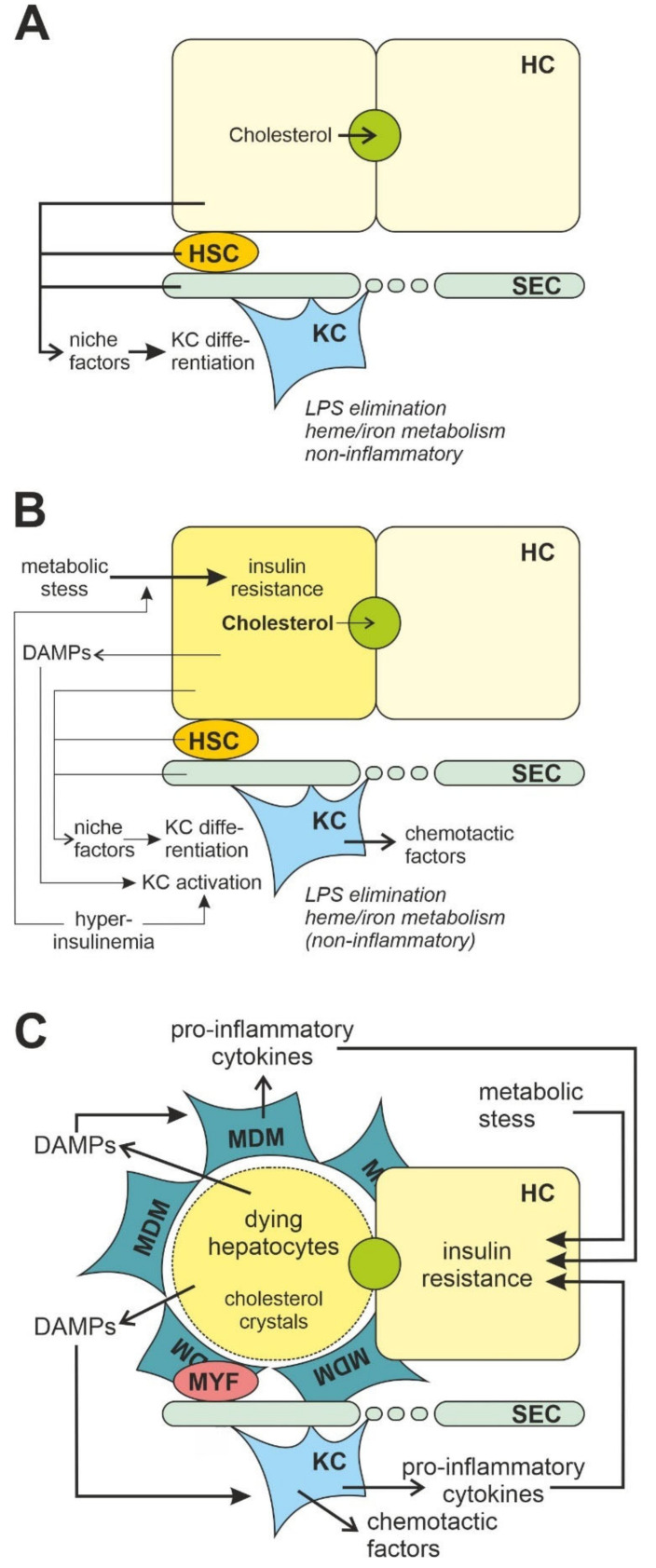
**Role of macrophages in the progression of hepatic insulin resistance.** (**A**) Normal liver. Functionally normal hepatocytes (HC), hepatic stellate cells (HSC) and sinusoidal endothelial cells (SEC) produce niche factors that maintain Kupffer cells (KC) in a differentiated, non-inflammatory state that allows them to perform their normal physiological function, among others, elimination of gut-derived LPS. Hepatocytes secrete excess cholesterol into the bile. (**B**) Transition state. This hypothetical intermediate state illustrates how an initial impairment of Kupffer cell function can trigger the ensuing low-grade inflammation. Increasing metabolic stress and hyperinsulinemia caused by peripheral insulin resistance increasingly impair HC functions. Reduced production of niche factors and hyperinsulinemia combined with enhanced exposure to activating cues such as danger-associated molecular pattern (DAMP) drive KC towards a more pro-inflammatory state and production of chemotactic factors. (**C**) Inflammation. Attracted by chemotactic factors of KC, monocyte-derived macrophages (MDM) form crown-like structures around dying hepatocytes that release large amounts of DAMPs. Excess cholesterol forms cholesterol crystals. DAMPs and cholesterol crystals activate both MDM and KC to produce pro-inflammatory cytokines that, in concert with metabolic stress, render HC increasingly insulin resistant. HSCs start to transdifferentiate into myofibroblasts (MYF). The non-resolvable inflammation is self-perpetuated by the secretion of chemotactic factors.

Apart from macrophage activation in adipose tissue, inflammation in skeletal muscle and liver also contribute to systemic insulin resistance. While the primary sites of macrophage-driven inflammation in skeletal muscle appear to be intermuscular fat depots and hence regions of ectopic adipose tissue formation, insulin resistance in the liver depends on the activation of resident liver macrophages, the Kupffer cells and newly recruited bone-marrow derived macrophages infiltrating the liver parenchyma [127] (Figure 2). Similar to elimination of adipose tissue macrophages, elimination of Kupffer cells by intravenous injection of liposome-encapsulated clodronate blunted hepatic insulin resistance induced by a short-term (three-day) high-fat diet in mice [128] without affecting the number of macrophages in adipose tissue. Similar results were obtained after feeding interventions with high-sucrose or high-fat diets for two or four weeks; the diet-induced hepatic steatosis, glucose intolerance and insulin resistance were prevented by gadolinium chloride-mediated Kupffer cell depletion [129,130].

This contrasts with findings in mice with persisting diet-induced obesity. Kupffer cells were depleted by intraperitoneal injection of clodronate liposomes in animals that had received a high-fat diet for 15 to 17 weeks. Three days after the treatment with clodronate liposomes, a significantly higher hepatic lipid accumulation, impaired hepatic insulin receptor signaling and a worsened homeostatic model assessment of insulin resistance (HOMA-IR) were observed that went along with an abrogation of diet-induced IL-10 production in these livers, indicating that Kupffer cells alternatively activated by the high-fat diet intervention might be the main source for this insulin-sensitizing cytokine [131]. In addition, the high phagocytotic capacity of Kupffer cells was discussed as a main factor in preventing further damage of the liver. According to this model, Kupffer cells might only play a limited immunoregulatory role by the release of pro-inflammatory mediators [54,132,133].

However, while Kupffer cells constitute the largest macrophage population in a healthy liver and act as sentinels, the composition changes dramatically in a damaged liver [54,134] (Figure 2). Kupffer cells release chemokines such as C-C-motif ligand 2 (CCL2, alternative name monocyte chemoattractant protein 1, MCP1), a ligand of chemokine C-C-motif receptor 2 (CCR2) and a dominant mediator, which causes massive infiltration of monocytes, especially Ly6C^high^ CCR2^+^ monocyte-derived macrophages with a pro-inflammatory signature in the liver [135,136,137,138]. Levels of CCL2 were increased in livers of NASH patients and murine models of NASH and fibrosis [135], and infiltration of Ly6C^high^ CCR2^+^ macrophages is considered a critical pathogenic event promoting the progression to steatohepatitis and insulin resistance [135,139,140]. Consistent with this, hepatic lipid accumulation and insulin resistance were decreased in the mouse model with diet-induced NAFLD when CD11c-positive cells were removed or animals were treated with a CCR2 antagonist [116,141].

Mediators released from Kupffer cells and infiltrating macrophages may affect glucose homeostasis in hepatocytes. Thus, Kupffer cells appear to be a major source for IL-1β in diet-induced obesity in mice and Kupffer cell-derived IL-1β contributed to the development of hepatic steatosis [142] and the ensuing insulin resistance. Reduction in the production of pro-inflammatory cytokines, among others IL-1β, selectively in Kupffer cells but not in other macrophages or hepatocytes by Kupffer cell-specific silencing of NFκB signaling using β1,3-d-glucan-encapsulated siRNA particles, improved insulin sensitivity in genetically obese mice on a high-fat diet [143]. A similar approach revealed that Kupffer cell cannabinoid 1 (CB1) receptors might be involved in the activation of Kupffer cells. Elimination of these receptors on Kupffer cells improved insulin sensitivity in diet-induced obese mice and reduced the expression of some, but not all pro-inflammatory cytokines [144]. Notably, the TNFα expression was not affected, although TNFα appears to play a pivotal role in the development of insulin resistance in vivo (see below).

Kupffer cells incubated with bovine serum albumin (BSA)-palmitate for 24 h expressed higher levels of the pro-inflammatory cytokines IL-6, TNFα and IL-1β, all of which are known to induce insulin resistance, than Kupffer cells incubated with BSA-oleate or BSA-control. By contrast, BSA-oleate induced the expression of IL-10. Supernatants of BSA-palmitate but not BSA-oleate-treated Kupffer cells in turn activated stress kinases and STAT3 signaling and impaired insulin-dependent Akt phosphorylation in primary hepatocytes [145]. Similarly, OSM produced in Kupffer cells in response to the lipid mediator prostaglandin E_2_ (PGE_2_), whose production is increased in the liver by a NASH-inducing diet [146], impaired insulin signaling in hepatocytes [147]. Along the same lines, an insulin-dependent induction of pro-inflammatory cytokines has been shown to impair insulin signaling in hepatocytes [87], indicating that the compensatory hyperinsulinemia, which ensues insulin resistance, may aggravate insulin resistance particularly in the liver, which—via the portal circulation—is exposed to much higher insulin concentrations than all peripheral organs.

Furthermore, there is some evidence that Kupffer cells and infiltrating macrophages in steatotic livers might also contribute to the whole body production of the adipokine resistin and thereby enhance hepatic insulin resistance [148].

Metabolically stressed hepatocytes [149] release, among others, the lipid mediator leukotriene B4 (LTB4) that acts as chemotactic factor to recruit macrophages. Upon local activation, Kupffer cells [150] and newly recruited macrophages can release additional LTB4. LTB4 in turn increases glucose output from hepatocytes and impairs insulin-dependent Akt phosphorylation and abrogates the insulin-dependent inhibition of glucagon-stimulated glucose formation [149] thereby contributing to insulin resistance and hyperglycemia. Other arachidonic acid-derived lipid mediators such as PGE_2_ produced in Kupffer cells and infiltrating macrophages in insulin-resistant livers plays an ambiguous role in the development of insulin resistance. While PGE_2_ can enhance the production by macrophages of a number of pro-inflammatory cytokines that cause insulin resistance [151,152], enhance the cytokine-induced insulin resistance in hepatocytes [153] and thereby contribute to the development of insulin resistance, it inhibits the production of TNFα in macrophages [154]. In vivo, the PGE_2_-dependent inhibition of the TNFα formation appears to outweigh the stimulation of other pro-inflammatory cytokines, because attenuation of diet-induced PGE_2_ formation by genetic deletion of the microsomal PGE synthase 1 (mPGES-1), a key enzyme in the synthesis of PGE_2_, enhanced the relative insulin resistance index in comparison to wildtype animals receiving the same diet [146].

## 5. Role of Macrophages in the Development of Insulin Resistance: Insulin Resistance as a Cause of Inflammation

In contrast to the concept presented in the previous section, several studies have shown that insulin resistance in adipose tissue precedes macrophage infiltration and inflammation, and some even claim that insulin resistance might be the cause rather than consequence of inflammation [155].

### 5.1. Adipose Tissue

Although there is good evidence that adipose tissue inflammation and in particular infiltration of adipose tissue with type I macrophages is a relevant driver in the development of insulin resistance, recent evidence challenges this view. Thus, it has been shown that in adipose tissue insulin resistance determined by the impairment of insulin-dependent suppression of lipolysis correlated with the size of adipocytes but not with the macrophage number in adipose tissue. Weight loss that improved insulin sensitivity was accompanied by a reduction in adipocyte size but not by a reduction in the macrophage number. Furthermore, there was no association between the expression of the pro-inflammatory cytokines TNFα, IL-6 or IL-1β and the insulin-dependent suppression of lipolysis as a parameter of insulin sensitivity after the adjustment for fat cell size [156].

Similarly, metabolic parameters improved after bariatric surgery already after three months, whereas it took six months in the same cohort of patients to achieve a significant improvement in the body mass index and a reduction in the circulating pro-inflammatory cytokine levels, among others TNFα, IL-1β and IL-6 [157]. Notably, circulating MCP1 levels did not significantly decrease during the observation period in this study.

Feeding a high-fat diet for only three days to C57BL/6J mice resulted in impaired glucose tolerance and insulin resistance. Although macrophage infiltration in adipose tissue of these mice was observed after this short-term intervention, adipose tissue macrophages appeared not to be essential for the development of glucose intolerance or insulin resistance, as mice with a clodronate-mediated >80% depletion of macrophages still became glucose intolerant and insulin resistant after three days of consuming a high-fat diet [158]. In the same study, levels of ceramides, diacylglycerol and non-esterified fatty acids were augmented in muscle and liver tissue after this short-term feeding, suggesting an acute lipotoxic mechanism as a main cause of insulin resistance. Lipid levels were not affected by treatment of clodronate to eliminate macrophages. Moreover, mice with lymphocyte deficiency were not protected from short-term high-fat-diet induced glucose intolerance, insulin resistances and accumulation of macrophages in the adipose tissue [158]. Conversely, intravenous lipid infusion in humans and rodents promoted skeletal muscle and systemic insulin resistance [159,160,161] that could be blunted by inhibition or genetic ablation of IKKβ or PKCθ [162,163].

A recent animal study showed that, rather than inflammation, the metabolic overload of the adipocyte might be responsible at least for the early phases of adipose tissue insulin resistance. A seven day high-fat diet feeding increased adipocyte plasma membrane 1,2-diacylglycerol content, activated PKCε and impaired insulin-dependent suppression of lipolysis supposedly by phosphorylation of Thr^1150^ in the insulin receptor β-chain, as mice with a Thr^1150^Ala substitution in the receptor were protected from insulin resistance [164]. A recent human study implied that adipose tissue insulin resistance measured by the insulin-mediated suppression of lipolysis could be predicted by fat cell size but was not related to adipose tissue inflammation [156]. In mice, enhanced lipolysis caused insulin resistance in the liver and skeletal muscle, whereas an acute reduction in fat cell lipolysis by suppression of adipocyte G_i_-signaling protected obese mice from insulin resistance and glucose intolerance [165].

Along the same lines, white adipose tissue-specific insulin resistance caused an increase in adipocyte MCP1 expression and an accumulation of macrophages in adipose tissue-specific TORC2 (Rictor-knockout mice, AdRiKO), in which insulin-dependent Akt-activation was impaired [155], hence arguing that insulin resistance precedes inflammation.

### 5.2. Liver

Although beyond any doubt activation of Kupffer cells and infiltrating macrophages contribute to the development of hepatic insulin resistance, it cannot be excluded that this inflammatory response is secondary to the release of activating factors, e.g., DAMPs, by hepatocytes under metabolic strain. In support of such a model dietary cholesterol is associated with the development of liver dysfunction [166,167] (Figure 2). Dietary cholesterol is delivered preferentially to the liver as chylomicron remnants. Depending on the systemic demand, it is either incorporated into VLDL particles for delivery to the periphery or excreted into bile either as free cholesterol or after conversion into bile acids. The capacity for excretion is, however, limited. Above a certain threshold, which in mice has been determined to be 0.5% cholesterol in the diet, cholesterol starts to accumulate in the liver [168]. An elevated cholesterol content in the ER membrane impaired the function of the sarco/endoplasmatic reticulum calcium ATPases (SERCA) calcium pump of the endoplasmic reticulum, which resulted in a luminal decrease in calcium concentration, impaired protein folding, and finally the development of ER stress. However, the contribution of this mechanism to hepatic inflammation and NASH is discussed controversially [167,169,170]. Furthermore, accumulation of cholesterol in the outer phospholipid membrane of lipid droplets impaired lipid transport and caused the formation of cholesterol crystals, which have been detected in both human NASH and murine NASH models [167,168,171,172]. Notably, excessive incorporation of cholesterol into mitochondrial membranes impaired the transport of glutathione from the cytosol to the mitochondria [173] and consequently increased oxidative stress [174]. Lipid peroxides and oxysterols also decreased the expression of mitochondrial respiratory chain complexes and disrupted mitochondrial function further promoting cellular stress conditions in hepatocytes [175]. Because ER stress and oxidative stress and mitochondrial dysfunction are linked in a vicious cycle, mitochondrial dysfunction can progress to an extent that glucose homeostasis gets impaired [176]. Cholesterol-derived oxysterols also antagonized Akt kinase-mediated anti-apoptotic (survival) signaling pathways [177] promoting the release of DAMPs from metabolic stressed hepatocytes.

Fatty acid infusion increased glucose output from hepatocytes and impaired insulin signaling without triggering an inflammatory response in animal models and humans [178,179,180,181]. Furthermore, insulin resistance can be elicited in isolated hepatocytes or hepatocyte-like cell lines by inducing metabolic stress in absence of other cell types, in particular macrophages [182]. Thus, ER stress induced by agents such as tunicamycin activated ER stress sensors such as protein kinase RNA-like endoplasmic reticulum kinase (PERK), activating transcription factor 6 (ATF4) and tribbles-like protein 3 (TRB3) in the liver [183] and rendered hepatocytes insulin resistant [182]. Knockdown of the inositol-requiring enzyme 1α (IRE1α) as a key trigger of the ER stress response prevented insulin resistance. Since ER stress renders hepatocytes more susceptible to cell death [176,184] subsequent activation macrophages by DAMPs from dying hepatocytes may further propagate insulin resistance. In vivo, the ER stress response and hepatic insulin resistance were observed in mice already after three days of high-fat diet feed before any signs of inflammation in the liver were visible. The rapid induction of insulin resistance was blunted by inhibiting the ER stress-dependent α-subunit of the eukaryotic initiation factor 2 (eIF2α) pathway [185].

Thus, both in vitro and in vivo evidence clearly shows that hepatic insulin resistance can be dissociated from inflammation. Thus, cholesterol or fatty acid overload causing metabolic stress and insulin resistance in hepatocytes might precede inflammation and inflammation would be a consequence rather than the cause of metabolic dysfunction.

How can these apparently contradicting results be reconciled? A likely explanation is the time course of the events. Whereas local insulin resistance and systemic glucose intolerance can be triggered by an acute metabolic overload in adipocytes already during a short period of excessive overfeeding, structural changes in adipose tissue and liver that accompany prolonged dietary calorie excess result in sustained low-grade inflammation that will increasingly contribute to systemic insulin resistance. Whereas metabolically triggered insulin resistance in response to an acute overload with lipids might be envisaged as a physiological response to allow appropriate lipid handling that is supported by the action of resident macrophages, the inflammatory response triggered by sustained over-nutrition is the first step into a self-amplifying vicious cycle that results in a progressive dysfunctionality of adipose tissue and liver in which inflammation contributes significantly to the maintenance and propagation of insulin resistance. In accordance with such an explanation, the type and the extent of the diet-induced inflammatory response varies between different phases of diet-induced obesity in mice [186].

## 6. Direct Modulation of Macrophage Function by Insulin

As outlined above, there appears to be a tight mutual interrelationship between macrophage-driven chronic low-grade inflammation and insulin resistance. Insulin resistance goes along with hyperinsulinemia. Macrophages express insulin receptors and healthy macrophages are responsive to insulin. Thus, two questions arise: how does insulin affect macrophage function, and could the insulin-dependent modulation of macrophage function be affected by hyperinsulinemia or insulin resistance in macrophages?

Several studies indicate that insulin may directly affect the inflammatory response in macrophages. While most of these studies suggest that insulin either triggers an inflammatory response or enhanced the secretion of pro-inflammatory cytokines (see below) others failed to see an increase in cytokine production [187] or even reported an anti-inflammatory potential of insulin.

### 6.1. Pro-Inflammatory Actions of Insulin

The observation that resident macrophages in the parenchyma of the pancreatic islet that are constantly exposed to high insulin concentration show a more activated phenotype expressing comparatively high levels of TNFα and IL-1β, indirectly supports the hypothesis that insulin might represent a pro-inflammatory stimulus [188], although IL-1β formation in islet macrophages is likely a physiological regulator of insulin secretion [189].

Very early studies showed that the phagocytic and bactericidal capacity was impaired in macrophages of alloxane-diabetic rats and could be enhanced by stimulation with insulin. Phagocytosis and the production of H_2_O_2_ in Bacillus Calmette-Guérin (BCG) -stimulated rat peritoneal macrophages of alloxane-diabetic rats were almost abolished and were rescued by insulin [190], indicating that insulin signaling in macrophages is essential to maintain a bactericidal response.

Similarly, peritoneal macrophages isolated from streptozotocin-diabetic (type 1 diabetic, low insulin) mice responded to a stimulation with lipopolysaccharide or interferon γ with a lower secretion of TNFα and IL-6 than macrophages of control mice. By contrast, the IL-4-elicited expression of arginase was higher in macrophages of diabetic animals. Treatment of the diabetic mice with insulin rescued the stimulation-dependent production of the pro-inflammatory cytokines and blunted the IL-4-dependent expression of arginase [191].

Indirect evidence comes from studies that showed in humans and mice that circulating pro-inflammatory cytokine levels or adipose tissue macrophage accumulation were enhanced by insulin. Thus, it was observed that the number of macrophages in adipose tissue and circulating levels of TNFα and IL-1β of type 2 diabetic patients increased with the onset of insulin therapy [192], however, the result was confounded by the concomitant weight gain in these patients. Similarly, circulating IL-6 and TNFα levels were increased during an euglycemic hyperinsulinemic clamp in healthy human males [193] and IL-6 gene expression was enhanced in adipose tissue but not in skeletal muscle. Insulin apparently enhanced the production of IL-6 by a low dose of LPS under similar conditions [194]. Hyperinsulinemic euglycemic clamps increased the concentration of IL-6 and IL-8 in interstitial micro-dialysis samples of adipose tissue of obese women [195]. Similarly, IL-6 and TNFα levels were acutely increased in young healthy male and female subjects during a hyperinsulinemic euglycemic clamp [196]. Interestingly, in non-diabetic overweight persons, starting TNFα levels were already elevated to values seen after 240 min of clamp in the young healthy group. In overweight persons, TNFα levels did not further increase during the clamp. In patients, there was a strong correlation between the expression of these cytokines in adipose tissue and fasting insulin, whereas there was only a weak correlation with body mass index [197].

In a hyperinsulinemic genetically obese mouse model, circulating cytokine levels were decreased by reducing insulin levels by streptozotocin treatment. By contrast, cytokine levels were increased in lean animals by insulin infusion [197].

It is assumed that the bulk of cytokines formed in adipose tissue does not originate in adipocytes but other “non-fat cells”, mainly macrophages [198]. However, in all studies cited above and other studies it cannot be ruled out that the insulin-elicited increase in adipose tissue inflammation or cytokine release was not due to a direct interaction with macrophages but rather an indirect one. Thus, it has been shown that insulin can increase the expression of cell adhesion molecules on endothelial cells [199,200] and thereby might increase macrophage recruitment into adipose tissue. Alternatively, increased cytokine production might either be due to an indirect activation of macrophages by mediators released from other cells in response to insulin or else the cytokines might be released from other cells.

In a human in vivo study, LPS-elicited IL-6 and to a lesser extent TNFα production as well as the counter-regulatory cortisol release were enhanced or prolonged by an euglycemic hyperinsulinemic clamp. While this might suggest an insulin-dependent activation of macrophages, which are the main source of IL-6 and TNFα in the circulation, the same study failed to show an increase in IL-6 or TNFα formation by insulin in non-stimulated or LPS-stimulated isolated whole blood cells [201], rather arguing in favor of an indirect mechanism.

By contrast, insulin increased TNFα expression and secretion in THP-1 macrophages and human monocyte-derived macrophages in an ERK1/2-dependent manner [202], clearly showing a direct impact of insulin on macrophages that was independent of other stimuli. This could indicate that hyperinsulinemia could be a trigger for cytokine-dependent insulin resistance. In support of such a hypothesis, insulin increased the formation of IL-1β in U937 macrophages and thereby impaired the insulin-dependent induction of glucokinase in a cell-based model of hepatic insulin resistance [87].

High-fat diets impair the gut barrier for endotoxins and result in elevated LPS concentrations in the blood of patients or animals consuming a high-fat diet [203,204,205]. As a consequence, an inflammatory response is elicited that might be worsened by hyperinsulinemia because insulin also enhances the LPS-stimulated expression and secretion of IL-1β and IL-8 from U937 macrophages or primary macrophages differentiated from human-blood mononuclear cells [151]. The insulin-dependent increase in cytokine production possibly was mediated in part by eliciting a prostaglandin E_2_-dependent positive autocrine feed-forward loop. Similarly, both insulin and prostaglandin E_2_ enhanced the palmitate-dependent IL-8 formation in THP-1 macrophages [152]. Similar results were found for the chemokine CCL2 (Klauder et al., unpublished results). This indicated that the combined presence of high insulin and palmitate concentrations that prevail in insulin-resistant tissue might act in concert to enhance the release of pro-inflammatory cytokines and chemokines from macrophages. At variance with such a hypothesis, the palmitate-dependent IL-1β formation in human monocyte-derived metabolically activated macrophages was not further enhanced by the simultaneous presence of high insulin and glucose concentrations [44].

Selective elimination of insulin receptor expression in myeloid cells by crossing insulin receptor gene floxed mice with mice expressing Cre recombinase under the lysozyme promoter provided insight into the direct action of insulin on macrophages in vivo. While no differences were observed between myeloid insulin receptor-deficient and control mice on a chow diet, on a high-fat diet myeloid insulin receptor-deficient mice became less insulin resistant despite a similar weight gain and body fat accumulation. Notably, while serum TNFα levels were elevated in wild-type animals, the increase in serum TNFα was blunted by the myeloid-specific insulin receptor ablation. The number of adipose tissue macrophages was reduced and the diet-dependent induction of TNFα and CCL3 in stromal vascular cells of the adipose tissue was blunted. There is, however, one major caveat to this model: The Cre expression is driven by the endogenous lysozyme promoter. Hence, insulin receptor deficient macrophages are at the same time heterozygous deletion mutants for lysozyme. The observed effects might therefore in part result from this additional difference. This concern is perhaps underscored by the observation of the authors that both basal- and palmitate-induced expression of matrix metalloproteinase 9 was attenuated in peritoneal macrophages of myeloid-specific insulin receptor-deficient animals in culture, independent of the presence of insulin [206]. Myeloid-specific deletion of the insulin receptor resulted in a reduced LPS-dependent IL-1β and IL-6 expression and reduced atherosclerosis in apolipoprotein E-deficient mice [207].

Along the same line, LPS-stimulated expression of TNFα, IL-1β and IL-6 was blunted in adipose tissue macrophages isolated from high-fat diet-fed mice with a myeloid-specific deletion of IRS2. However, IRS2 in myeloid cells is not only part of the insulin and IGF receptor signal chain, but is also involved in IL-4 signaling [208]. Yet, the interruption of the anti-inflammatory IL-4 receptor signal chain by an IRS2 knockout should rather enhance and not attenuate the LPS-dependent production of pro-inflammatory cytokines.

### 6.2. Anti-Inflammatory Actions of Insulin

In mouse peritoneal macrophages or RAW264.7 mouse macrophages that were maintained in presence of high glucose, insulin increased the expression of IL-10 and at the same time decreased the expression of TNFα, IL-1β and iNOS [209]. These effects of insulin were abrogated by prior incubation of the cells with either MEK inhibitor U0126 or PI3K inhibitor wortmannin. This is in accordance with the observation that constitutive activation of PI3K in alveolar macrophages of HIV patients resulted in an enhanced IL-10 and decreased TNFα production [210]. Similarly, LPS-stimulated PI3K and Akt activity increased in Balb/c mice with age. The increase was accompanied by a decrease in toll-like receptor-dependent formation of TNFα, IL-6 and IL-12 and an increase in IL-10 formation. These alterations were reverted by inhibition of PI3K [211].

In one study, pre-treatment of THP-1 macrophages with insulin for 24 h enhanced the LPS-dependent Akt phosphorylation but delayed the MAPK phosphorylation and attenuated the NFκB activation. This resulted in an attenuation of the production of TNFα and IL-8. The authors hypothesized that insulin, by inducing an activin A-dependent autocrine loop, induced src-homology inositol phosphatase (SHIP) activity that retarded or blunted the MAPK or NFκB dependent cytokine induction [212]. This hypothesis is, however, at odds with a different study that showed that LPS-stimulated cytokine production was mitigated by ablation and NFκB activation was enhanced by over-expression of SHIP [213]. Thus, an insulin-dependent induction of SHIP would have been expected to enhance the LPS-dependent cytokine production.

In RAW264.7 cells that were transformed into cholesterol-laden foam cells, insulin attenuated the expression of TNFα and IL-6 likely by downregulation of MyD88 that resulted in a lesser activation of NFκB. These effects were inhibited by wortmannin and hence were assumed to be mediated by activation of the PI3K-Akt pathway [214].

In LDL receptor-deficient mice that were whole-body irradiated and then transplanted with bone marrow cells of insulin receptor-deficient mice, more apoptotic foam cells were detected in atherosclerotic lesions. In vitro, peritoneal macrophages isolated from insulin receptor-deficient mice were more susceptible to the induction of apoptosis by oxidized LDL or 2-deoxy-glucose, both inductors of ER stress. These data might indicate that proper insulin signaling is essential to allow tissue macrophage survival in the phase of inflammation resolution [215].

Insulin can activate anti-apoptotic signal chains and enhance macrophage survival. Thus, it attenuated the LPS-induced apoptosis in THP-1-macrophages [216]. In addition, insulin decreased LPS-induced TNFα and IL-1β production in THP-1 macrophages. However, excessively high concentrations of LPS (up to 50 µg/mL) were used in this study.

In conclusion, the impact of insulin on macrophage cytokine release appears to depend on the origin and the prior state of activation of macrophages. This assumption is supported by direct evidence: While insulin enhanced the LPS-dependent production of IL-6 and TNFα in bone marrow-derived macrophages from diabetic mice by a PI3K/ERK-dependent mechanism, it inhibited the LPS-dependent secretion of TNFα and IL-6 from the alveolar macrophages of these mice [217,218]. On the other hand, insulin increased the phagocytotic activity of alveolar macrophages from diabetic rats apparently by a direct activation of PKCδ [219].

In THP-1 macrophages at low physiological glucose concentrations (5.5 mM), insulin enhanced the expression of the M1-typical marker iNOS, whereas at high glucose concentrations (25 mM) the already elevated expression of iNOS was significantly repressed and the glucose-dependent repression of the M2 marker CD206 was attenuated by insulin [220]. The anti-inflammatory effect of insulin was mediated by an Akt-dependent induction and activation of peroxisome proliferator activated receptor γ and attenuation of the glucose-dependent STAT1 activation.

### 6.3. Impaired Macrophage Function Due to Macrophage Insulin Resistance

Since macrophage function apparently is directly regulated by insulin, it may be assumed that insulin resistance in macrophages also affects their function. There are a couple of examples in support of this notion:

FoxO1 expression and IL-1β expression was elevated in macrophages of insulin-resistant db/db mice in comparison to heterozygous controls. A FoxO1 response element was identified in the IL-1β promoter. In RAW264.7 cells transfection with an expression vector for FoxO1 increased the activity of a luciferase under the control of the IL-1β promoter. This increase was blunted by the addition of insulin. The authors interpreted the results in this rather artificial system as evidence that insulin-resistance in macrophages might favor an inflammatory response [221].

Insulin in concert with LPS stimulates the production IL-10, which in turn enhances the insulin sensitivity of the surrounding metabolically active cells. The insulin-dependent IL-10 production was abrogated by Akt1 or Akt2 knockout and was dependent on a mTOR activation. Insulin resistance in macrophages thus might result in impaired IL-10 formation and thereby contribute to worsening systemic insulin resistance [88].

Hyperinsulinemia also results in a downregulation of endogenous IRS2 in macrophages. Apart from the resulting insulin resistance, the impairment of other signaling chains that rely on IRS2 may contribute to the alteration of macrophage functions. Thus, it has been shown that a downregulation of IRS2 by hyperinsulinemia-mediated signaling impaired the polarization of macrophages by the IL-4/IRS2/Akt pathway due to the stabilization of a FoxO1-dependent corepressor complex [222,223].

Macrophages previously exposed to LPS can become refractory to a subsequent stimulation. This endotoxin tolerance is dependent on the activation of Akt1 resulting in suppression of the microRNA miR-155, an increase in let-7e and downregulation of TLR4 [224,225]. In endothelial cells, miR-155 was repressed by insulin and insulin resistance due to impaired signaling by a variant of IRS1 (Arg^972^ IRS1) resulting in an upregulation of miR-155 [226]. miR-155 deficient mice show no glucose intolerance or insulin resistance despite exacerbated obesity and apparent MAFLD [227]. Although no studies seem to exist that directly analyze the regulation of miR-155 in macrophages by insulin, it appears possible that a dysregulation of miR-155 expression in insulin-resistant macrophages might favor a stronger pro-inflammatory response to TLR4 activation.

## 7. Concluding Remarks

**Figure 3:**
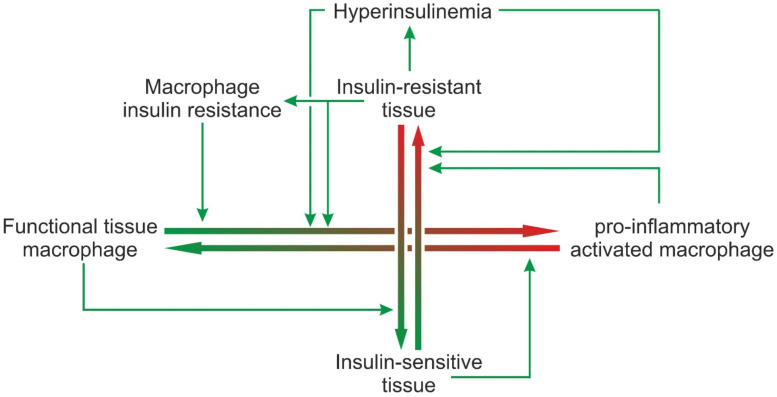
**Schematical overview of the mutual interrelation between macrophage differentiation, tissue insulin resistance and hyperinsulinemia.** Pro-inflammatory macrophage activation, tissue insulin resistance and hyperinsulinemia are mutually interrelated and may promote each other in vicious feed-forward cycles (green to read arrows). On the other hand, insulin-sensitive tissues provide cues to shape functional tissue macrophages which in turn provide signals that enhance tissue insulin sensitivity (red to green arrows). For details see concluding remarks.

In normal tissue homeostasis, tissue macrophages contribute to the maintenance of functional tissue integrity. At the same time, cues released from healthy tissue are essential to maintain or achieve the phenotype of a fully functional tissue macrophage. Tissue functions are regulated by physiological levels of insulin and the tissue as well as the macrophages being insulin sensitive. If any of the parameters are shifted to “pathological”, the system dis-equilibrates, and the disequilibrium is gradually worsened by feed-forward vicious cycles. If, for example, a metabolic overload renders metabolic active tissues such as white adipose tissue or liver insulin-resistant, this will elicit signals that will shift the equilibrium between functional tissue macrophages and pro-inflammatory activated macrophages towards the latter (Figure 3). Pro-inflammatory activated macrophages in turn will release cytokines that aggravate insulin resistance. In addition, the pancreas will try to compensate for insulin resistance by increased insulin production resulting in hyperinsulinemia. While physiological insulin concentrations probably help to maintain a functional tissue macrophage phenotype and to support PI3K-dependent inhibitory feedback loops that limit a pro-inflammatory response to PAMPs or DAMPs, hyperinsulinemia appears to enhance the effect of pro-inflammatory stimuli. Furthermore, tissue macrophages that are overloaded with debris from non-functional and dying tissue cells become insulin resistant and no longer react to the anti-inflammatory and anti-apoptotic insulin signal. Dying cells and accumulating debris will elicit chemotactic signals resulting in the recruitment of mononuclear cells from the circulation. Thereby, macrophage insulin resistance and hyperinsulinemia shift the equilibrium further towards pro-inflammatory activated macrophages. The ensuing chronic inflammation again contributes to insulin resistance (Figure 3).

The “egg-and-hen” problem is not finally solved and the exact sequence of the events in the development of inflammation and insulin resistance remains elusive, however several lines of evidence support the view that a local metabolic-stress response precedes the inflammatory response of macrophages. It is currently not known if one of the described impairments could serve as an isolated initial trigger or if the derangements rather develop in parallel. At later stages, however, metabolic stress, insulin resistance and inflammation augment each other mutually in vicious feed-forward cycles.

Future research should aim to better characterize the response of different populations of macrophages isolated from healthy and metabolically inflamed tissues to physiological and pathological cues, in particular normal and elevated levels of insulin and various metabolites. It will be difficult to address this issue experimentally due to the high phenotypic flexibility of macrophages that will rapidly change their properties after removal from the local environment. In situ studies, on the other hand, are hampered by the enormous complexity of the local signaling networks in the tissue. Since tissue-resident macrophages are key players in controlling normal tissue homeostasis and deviations from the normal phenotypic profile enhances metabolic tissue dysfunction, it will be important to characterize the cues and signaling chains that contribute to tissue-specific differentiation in more depth and to identify potential targets to either prevent or reverse the pathological differentiation of tissue macrophages to a more inflammatory phenotype that contributes to the development of insulin resistance.

## Data Availability

Not applicable.

## Abbreviations

AS160: Akt target of 160 kDa; BCG: Bacillus Calmette-Guérin; BSA: bovine serum albumin; C3aR, C5aR: complement anaphylatoxin C3a and C5a receptors; CCL2: C-C-Motif ligand 2; CCR2: C-C-motif receptor 2; CD: cluster of differentiation; DAMPs: damage-associated molecular pattern: EMR1: Epidermal growth factor-like module containing mucin-like hormone receptor 1; ER: Endoplasmatic reticulum; ERK1/2: extra-cellular signal-regulated kinases 1/2; FoxO: Forkhead box protein O; Glut4: glucose transporter 4; GSK3β: Glycogen synthase kinase 3β; HIF1α: hypoxia-induced factor 1α; HOMA-IR: Homeostatic Model Assessment of Insulin Resistance; IFNRA: interferon receptor α; IFNRG: interferon receptor γ; IFNγ: interferon γ; IKK: inhibitor of kappa B kinase; IL: Interleukin; iNOS: inducible nitric oxide synthase; IRAKs: IL-1 receptor-associated kinases; IRS: insulin receptor substrates; IκB: inhibitor of kappa B; JAK: janus kinase; JNK: c-Jun N-terminal kinase; LDL: low-density lipoprotein; LAM: lipid-associated macrophages; LPS: lipopolysaccharide; LTB4: leukotriene B4; Ly6C: lymphocyte antigen complex locus C; Lyve1: lymphoid endothelium hyaluronan receptor 1; MAFLD: metabolic dysfunction-associated fatty liver disease; MAPK: mitogen-activated kinases; MCP1: monocyte chemoattractant protein 1, similar to CCL2; MEK: mitogen-activated protein kinase; MHCII: major histocompatibility complex II; MIF: macrophage migration inhibitory factor; mPGES1: microsomal prostaglandin E synthase 1; mTOR: molecular target of rapamycin; MyD88: myeloid differentiation primary response 88; NADPH: nicotinamide adenine dinucleotide phosphate; NAM: NASH-associated macrophages; NFκB: nuclear factor kappa B; PAMPs: pathogen-associated molecular pattern; PI2: 4,5-phosphatidyl-inositol-bisphopshate; PI3: 3,4,5-phosphatidylinositol-trisphosphate; PI3K: phosphorinositide-3-kinases; PKB/Akt: protein kinase B; PKC: protein kinase C; PGE_2_: Prostaglandin E_2_; SHIP: Src-homology inositol phosphatase; siRNA: small interfering ribonucleic acid; SOCS: suppressors of cytokine signaling; STATs: signal transducers and activators of transcription; TAB: TGF-β-activated kinase binding protein; TAK: TGF-β-activated kinase; TGFβ: Transforming growth factor β; Timd4: T cell immunoglobulin and mucin domain containing 4; TIR: Toll/interleukin-1 receptor domain; TIRAP: TIR domain–containing adapter protein; TLR: toll-like receptor; TNFα: tumor necrosis factor α; TORC: target of rapamycin complex; TRAF6: TNF receptor-associated factor 6; TRAM: TRIF-related adaptor molecule; TREM2: triggering receptor expressed on myeloid cells 2; TRIF: TIR-domain containing adapter-inducing interferon β; TSC2: tuberous sclerosis complex 2; Tyk2: tyrosine kinase 2; VAM: vascular-associated macrophages.
